# Selective quantification of the 22-kDa isoform of human growth hormone 1 in serum and plasma by immunocapture and LC–MS/MS

**DOI:** 10.1007/s00216-022-04188-z

**Published:** 2022-07-15

**Authors:** Bas Sleumer, Martijn van Faassen, Michel J. Vos, Rainer Bischoff, Ido P. Kema, Nico C. van de Merbel

**Affiliations:** 1 ICON Bioanalytical Laboratories, Amerikaweg 18, 9407 TK Assen, The Netherlands; 2 Department of Analytical Biochemistry, University of Groningen, A. Deusinglaan 1, 9700 AV Groningen, The Netherlands; 3 Department of Laboratory Medicine, University of Groningen, University Medical Center Groningen, EA61, P.O. Box 30.001, 9700 RB Groningen, The Netherlands

**Keywords:** Human growth hormone, Liquid chromatography-tandem mass spectrometry (LC–MS/MS), Biomarker, Immunocapture, Isoform

## Abstract

The human growth hormone GH1 (22 kDa) is a commonly measured biomarker for diagnosis and during treatment of growth disorders, but its quantification by ligand binding assays may be compromised by the occurrence of a number of isoforms. These can interfere in the assays and lead to differences in results between laboratories and potentially even in the treatment of patients. We present an LC–MS/MS method that is able to distinguish the major growth hormone isoform (GH1, 22 kDa) from other isoforms and quantify it without any interference across the clinically relevant concentration range of 0.5 to 50 ng/mL. Analysis involves purification of a 100-µL serum sample by immunocapture using an anti-GH-directed antibody, tryptic digestion, and LC–MS/MS quantification of an isoform-specific signature peptide for GH1 (22 kDa). A tryptic peptide occurring in all GH isoforms is monitored in the same 16-min analytical run as a read-out for total GH. Stable-isotope-labeled forms of these two peptides are included as internal standards. Full validation of the method according to recent guidelines, against a recombinant form of the analyte in rat plasma calibrators, demonstrated intra-assay and inter-assay imprecision below 6% across the calibration range for both signature peptides and recoveries between 94 and 102%. An excellent correlation was found between nominal and measured concentrations of the WHO reference standard for GH1 (22 kDa). Addition of up to 1000 ng/mL biotin or the presence of a 100-fold excess of GH binding protein did not affect the measurement. Equivalent method performance was found for analysis of GH in serum, EDTA, and heparin plasma. Analyte stability was demonstrated during all normal sample storage conditions. Comparison with the IDS-iSYS GH immunoassay showed a good correlation with the LC–MS/MS method for the isoform-specific signature peptide, but a significant positive bias was observed for the LC–MS/MS results of the peptide representing total GH. This seems to confirm the actual occurrence of other GH isoforms in serum. Finally, in serum from pregnant individuals, no quantifiable GH1 (22 kDa) was found, but relatively high concentrations of total GH.

**Graphical abstract:**

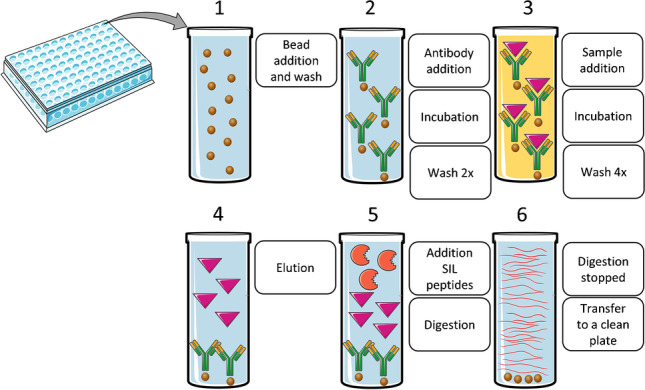

**Supplementary Information:**

The online version contains supplementary material available at 10.1007/s00216-022-04188-z.

## Introduction

**Fig. 1:**
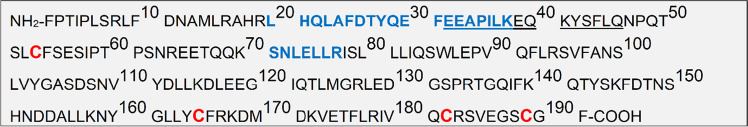
The 191-amino-acid sequence of the GH1 22-kDa isoform. The sequence indicated by the underlined amino acids is deleted in the GH1 20-kDa isoform. The cysteines at positions 53 and 165 and at positions 182 and 189 are connected by a disulfide bridge. The blue-colored peptides are selected as the signature peptides

**Fig. 2:**
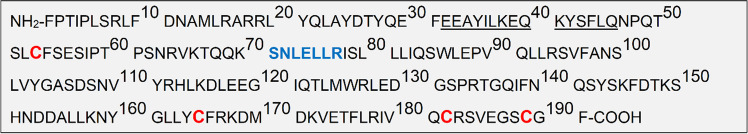
The 191-amino-acid sequence of the GH2 22-kDa isoform. The sequence indicated by the underlined amino acids is deleted in the GH2 20-kDa isoform. The cysteines at positions 53 and 165 and at positions 182 and 189 are connected by a disulfide bridge. The blue-colored peptide is selected as the signature peptide

Human growth hormone (GH) is a heterogeneous, endogenous protein, consisting of different variants, which are encoded by the *GH1* and the *GH2* genes on chromosome 17. *GH1* is expressed in the pituitary and *GH2* in the placenta. The main human growth hormone isoform (GH1, 22 kDa), a single-chain protein of 191 amino acids with two disulfide bridges, is derived from *GH1* and represents between 80 and 90% of total circulating GH [1–3]. A 20-kDa pituitary GH1 isoform is the second most abundant variant. It has a structure similar to the 22-kDa isoform, but lacks 15 of the latter’s amino acids and thus consists of 176 amino acids (Fig. 1). This form represents about 10% of circulating GH [1, 3]. Other minor GH1 variants, formed by alternative splicing, have also been identified [1, 4]. The GH1 isoforms occur in vivo in the free form as well as bound to growth hormone–binding proteins (GHBPs), and as covalent and non-covalent dimers [1]. GH1 and GH2 bind with equal affinity to GHBP. The main circulating isoform of placental GH2 is a 22-kDa single-chain protein with 191 amino acids and two internal disulfide bridges. It shows strong structural similarities with the major GH1 variant, but is a more basic protein [1–3] (Fig. 2). In addition, there is a 20-kDa GH2 with the same structure as the 22-kDa isoform but lacking 15 amino acids (Fig. 2). During pregnancy, GH2 is released into the maternal circulation with the highest concentrations in blood being reached in the last weeks of gestation. Together with an increased IGF-1 concentration, this results in the suppression of the maternally expressed GH1 isoforms [2]. The asparagine at position 140 in GH2 may be prone to glycosylation, which could result in the in vivo occurrence of a glycosylated and a non-glycosylated form [1–3]. Next to these GH forms, another structurally related endogenous protein exists, human chorionic somatomammotropin (HCS), which is a placental hormone that is only present during pregnancy, with the highest maternal serum concentrations near term [5].

GH1 is a commonly measured biomarker in the diagnosis and during treatment of growth disorders, but because of the occurrence of many closely related isoforms, its quantification is far from straightforward. Traditionally, GH is measured with ligand binding assays (LBAs), which are based on the recognition of the protein by one or more binding reagents such as polyclonal or monoclonal antibodies. Although their popularity is understandable because of their sensitivity, high sample throughput, and relatively low cost, for most commercially available GH assays, the specificity of their reagents is unknown and it is thus unclear which different isoforms an LBA actually binds and, if so, to what extent. As a result, one of the key problems in GH measurement is the inter-manufacturer variability caused by differences in the recognition of the different isoforms of GH. Older assays typically use polyclonal antibodies which are able to capture the different GH isoforms at least to some extent, with varying affinity, resulting in concentrations representing a total GH concentration. The more recently used automated routine LBAs are based on monoclonal antibodies which are likely to measure just one or a few GH isoforms and thus report lower concentrations. In addition, the use of different reference materials may affect the outcome of the measurement of GH. The previous World Health Organization (WHO) reference standards 66/217 and 80/505 were made of pituitary extracts and contain a mixture of different GH isoforms with unknown proportions. The currently used WHO 88/624 and 98/547 reference standards are prepared by recombinant technologies and contain only the GH1 22-kDa isoform. It is the combination of reference standard and antibody that will eventually determine the response that is obtained by a certain LBA, and it thus also determines what the measurement result actually represents. An assay employing recombinant 22-kDa GH1 as reference standard and a highly specific antibody against this isoform is most likely to provide concentrations of just 22-kDa GH1. Other combinations will reflect a sum of different isoforms, the magnitude of which will depend on how well the different isoforms are recognized by the antibodies used [3]. On top of this, two commonly known interferences exist: pegvisomant and GHBP. Pegvisomant is a modified form of GH, which is used as a drug in the treatment of acromegaly and can potentially be recognized by an LBA, resulting in both negative or positive bias of GH concentrations [6, 7]. GHBP is a more general problem as elevated concentrations may compete with GH binding to the LBA reagent, lowering the response for GH [4, 7, 8].

All these factors influence the GH measurement result and, potentially, the clinical conclusions and diagnosis of GH deficiency or acromegaly, which are based on a GH concentration being below or above a certain cut-off value. Comparing results between laboratories that use different LBAs is therefore difficult, and even changing to a new lot of reference material or antibody reagent within the same laboratory can mean that the previous cut-off level has to be reconsidered. Liquid chromatography coupled to tandem mass spectrometry (LC–MS/MS) may be able to address the issue of LBA reagent variability. The technique has now been firmly established as the first choice for quantification of small-molecule drugs and biomarkers in biological matrices. In addition, it is seeing increased use for protein analytes, typically in combination with an enzymatic digestion to convert the protein of interest into a set of peptides, which are subsequently measured as a surrogate for the intact protein [8]. By quantifying one or more peptides that are unique for a specific GH isoform, a high and constant selectivity can be obtained for this isoform. So far, the experience with LC–MS for GH quantification has been limited. Two published methods target the recombinant form of GH1 (22 kDa), called somatropin, and these methods were developed for supporting preclinical pharmacokinetic studies in rats [9, 10]. Two other methods were described for the GH quantitative analysis in human plasma or serum. These, however, have lower limits of quantification above approximately 10 ng/mL, which is insufficient to cover the complete endogenous GH range [11, 12]. The published LC–MS/MS methods that do have quantification limits at the relevant low– to sub–nanogram per milliliter concentrations are specific for the 22-kDa form of GH1, but less useful for routine clinical analysis, because of their long run times (up to 180 min per sample), and/or less attractive because of the relatively large sample volumes required (500–800 µL) [13, 14].

In this paper, we describe an LC–MS/MS method for the absolute quantification of the GH1 22-kDa isoform in human serum and plasma over the relevant concentration range of 0.5 to 50 ng/mL. It includes a simultaneous, semi-quantitative readout for total GH. With a sample volume of 100 µL and an analytical run time of 16 min, this method is well suited for more routine application in specialized laboratories. In the described method, GH1 (22 kDa) and other isoforms are enriched by immunocapture using a monoclonal capture antibody. After trypsin digestion of the extracted proteins, two surrogate peptides are used for quantification: a unique peptide for the quantification of GH1 (22 kDa) and a second peptide, occurring in all isoforms, for determination of total GH. The method was validated extensively according to the most recent guidelines [15, 16] and compared with a routine clinical analyzer (IDS-iSYS) using anonymized clinical samples.

## Materials and methods

### Chemicals and materials

Growth Hormone Human Recombinant (GH1 22 kDa) (UniprotKB – P01241) (Cat. No. CYT-202), Growth Hormone Pituitary 20 kDa Human Recombinant (GH1 20 kDa) (Cat. No. CYT-259), Growth Hormone Placental 22 kDa Human Recombinant (GH-2 22 kDa) (Cat. No. CYT-235), Growth Hormone Placental 20 kDa Human Recombinant (Cat. No. CYT-337), and GHBP Human Recombinant (Cat. No. CYT-238) were purchased from Prospec Protein Specialists (Ness-Ziona, Israel). WHO International Standard Somatropin (Recombinant DNA-Derived Human Growth Hormone) (Cat. No. CYT-202) (NIBSC code: 98/574) was obtained from NIBSC (Hertfordshire, UK). Formic acid ≥ 95% (Cat. No. F0507), ammonium bicarbonate BioUltra ≥ 99.5% (Cat. No. 9830), bovine serum albumin (Cat. No. A9647), TWEEN20 (Cat. No. P5927), trypsin from porcine pancreas (crude) (Cat. No. T0303), trypsin from bovine pancreas (L-1-tosylamide-2-phenylethyl chloromethyl ketone (TPCK) treated) (Cat. No. T1426), trypsin from porcine pancreas (proteomics grade) (Cat. No. 6567), citric acid monohydrate (Cat. No. 33114), and the protein biotin labeling kit (Cat. No. 11418165001; Roche) were purchased from Sigma-Aldrich (St Louis, MO, USA). n-Dodecyl-β-D-maltoside (DDM) (Cat. No. 89903) and Pierce™ streptavidin magnetic beads (a slurry of 10 mg/mL, Cat. No. 88817) were obtained from Thermo Fisher Scientific (Waltham, MA, USA). Gibco Dulbecco’s phosphate-buffered saline (10 ×) (Cat. No. 14200–067) was obtained from Life Technologies Europe B.V. (The Netherlands). Milli-Q water was prepared using a water purification system from Merck-Millipore (Burlington, MA, USA). Acetonitrile was purchased from Biosolve (Valkenswaard, The Netherlands). Custom-synthesized internal standard peptides SNLELLR with ^13^C_6_^15^N_4_-labeled C-terminal arginine and LHQLAFDTYQEFEEAYIPK with ^13^C_6_^15^N_2_-labeled C-terminal lysine were obtained from JPT Peptide Technologies (Berlin, Germany). The capture antibody, mouse anti-human growth hormone monoclonal antibody (Cat. No. ab9821), was purchased from Abcam (Cambridge, UK). Human serum, including samples from pregnant volunteers, and rat EDTA plasma were purchased from BioIVT (West Sussex, UK). The University Medical Center Groningen (UMCG) (the Netherlands) provided fresh human serum and plasma from healthy individuals for stability and cross-over testing and anonymized leftover clinical patient samples for comparison with the GH immunoassay.

### Preparation of calibration and quality control samples

A 200-µg/mL stock solution of recombinant human growth hormone (GH1, 22 kDa) was prepared by dissolving the content of the vial of lyophilized protein (label claim: 200 µg) in 1.00 mL of water, containing 0.1% BSA and 0.1% DDM to enhance solubilization. This stock was used for the preparation of the calibration and quality control samples. A second stock solution (WHO International Standard Somatropin) was prepared at a concentration of 1.00 mg/mL by dissolving the lyophilized protein (label claim: 1.95 mg) with 1.95 mL water and used for the preparation of the quality control samples for the determination of method accuracy. The stocks were divided into 0.2-mL (WHO) and 0.1-mL (Prospec) aliquots in Eppendorf (Hamburg, Germany) Protein Lobind tubes and stored at − 80 °C. Two sets of standard solutions in rat plasma at 1.00 and 10.0 µg/mL were prepared freshly before use. The first set was used to prepare calibration samples in rat plasma at 0.500, 1.00, 2.50, 5.00, 10.0, 25.0, 40.0, and 50.0 ng/mL. Quality control samples were unspiked human serum, containing a low endogenous GH level, and the same human serum lot spiked with an additional 10.0 (medium level), 35.0 (high level), or 80.0 (integrity of dilution) ng/mL GH1 (22 kDa) using the second set of standard solutions. For accuracy testing, additional quality control samples were prepared in rat plasma at concentrations of 2.00, 10.0, and 35.0 ng/mL. All calibration and quality control samples were prepared in polypropylene tubes and stored at − 80 °C.

### Sample pretreatment

#### Biotinylation of the capture antibody

The anti-GH capture antibody was biotinylated using the protein biotin labeling kit. A volume of 500 µL of the antibody (1 mg/mL in PBS, pH 7.2, carrier free) was incubated with 22.0 µL freshly prepared biotin-7-NHS labeling solution (3 mM in DMSO) for 120 min at room temperature and 600 rpm and protected from light. After biotinylation, the remaining non-reacted biotin-7-NHS was removed using a Sephadex G-25 gel filtration column. The concentration of the biotinylated capture antibody was determined using the Implen NanoPhotometer® N120 (München, Germany) at 280 nm against a corresponding blank solution and found to be 173 µg/mL. The biotinylated antibody solution was divided into 0.1-mL aliquots in Eppendorf Protein Lobind tubes and stored at − 80 °C.

#### Immunocapture procedure

Aliquots of 10 µL streptavidin-coated magnetic beads were added to an Eppendorf Protein Lobind 500 µL 96-well plate and washed twice with 200 µL 0.1% TWEEN20 in 1 × PBS. In this and all following washing and incubation steps, the magnetic beads were isolated by letting the plate stand for 5 min on a 96-well magnet plate from Alpaqua Magnum FLX (Beverly, MA, USA) and removing the wash solution using a BenchTop Pipettor from Sorenson Bioscience Inc. (Murray, UT, USA). Next, aliquots of 100 µL freshly diluted biotinylated capture antibody solution (4.00 µg/mL) in 0.1% TWEEN20 in 1 × PBS were added. The samples were incubated at 37 °C and 900 rpm for 120 min using an Eppendorf Thermomixer® Comfort to allow binding of the biotinylated capture antibody to the streptavidin-coated magnetic beads. Next, the magnetic beads were isolated and washed twice with 200 µL 0.1% TWEEN20 in 1 × PBS. Aliquots of 100 µL of plasma or serum were then added and incubated at 37 °C and 900 rpm for 120 min to allow binding of human growth hormone to the bead-capture antibody complex. Next, the magnetic beads were isolated and washed twice with 300 µL 0.1% TWEEN20 in 1 × PBS and twice with 1 × PBS. After overnight storage at + 4 °C, GH was eluted off the beads with 25 µL 0.1 M citric acid solution in water:acetonitrile (90:10, v/v) for 10 min at 45 °C and 900 rpm.

#### Digestion procedure

To the eluate (including the beads), 25 µL of 250 mM ammonium bicarbonate (ABC) buffer was added. Next, 25 µL of trypsin (60 µg/mL in 250 mM ABC buffer) was added followed by 10 µL of the internal standard peptides (20 ng/mL of stable-isotope-labeled (SIL) LHQLAFDTYQEFEEAYIPK and 8 ng/mL of SIL SNLELLR) in a 70:30 (v/v) mixture of water and acetonitrile. After the addition of 10 µL of acetonitrile, the samples were digested at 37 °C and 900 rpm for 6 h using an Eppendorf Thermomixer® Comfort. The digestion was stopped by the addition of 10 µL of 10% aqueous formic acid and briefly vortex-mixed. Next, the magnetic beads were isolated by letting the plate stand for 5 min on a 96-well magnet plate and the samples were transferred to a clean Waters QuanRecovery 700-µL 96-well plate using a BenchTop Pipettor, sealed, and placed in an autosampler at 10 °C for analysis.

### Chromatography and detection

The processed samples were injected into an M-class UPLC system coupled to a Xevo TQ-S triple-quadrupole mass spectrometer (Waters, Milford, MA, USA). Chromatographic separation was performed at 60 °C on a 100 × 1.0 mm (particle size 1.6 µm, pore size 100 Å) Luna Omega C18 column (Phenomenex, CA, USA). Mobile phase A consisted of 0.1% formic acid in water, and mobile phase B was 0.1% formic acid in acetonitrile. Gradient elution was performed using the following profile at a flow rate of 80 µL/min: 0.0–6.0 min: 13–16% B; 6.1–11 min: 20–25% B; 11.1–14: 95% B; and 14.1–16 min: 13% B. The injection volume was 8 µL. The mobile phase was diverted to waste between 0 and 3 min and between 11.5 and 16 min.

Detection of the two signature peptides, SNLELLR for total GH and LHQLAFDTYQEFEEAYIPK for GH1 (22 kDa), was performed in positive electrospray ionization mode. System operation and data acquisition were controlled using Waters MassLynx 4.1 and data was processed using TargetLynx 4.1 software. The following general instrument parameters were used: capillary voltage: 3000 V; source offset: 60 V; desolvation temperature: 400 °C; cone gas flow: 150 L/h; desolvation gas flow: 800 L/h; collision gas flow: 0.15 L/min; and nebulizer gas flow: 3 bar. Peptide-specific parameters are presented in Table 1.

**Table 1:** Detection parameters for the signature peptides

Peptide	Q1 *m*/*z*	Q3 *m*/*z*	Cone voltage (V)	Collision energy (V)
SNLELLR^a^	422.7 [M + H]^+^	530.3 (y4^+^)	20	14
SNLELLR^b^	422.7 [M + H]^+^	643.4 (y5^+^)	20	14
SNLELLR-SIL^a^	427.8 [M + H]^+^	540.3 (y4^+^)	20	14
SNLELLR-SIL^b^	427.8 [M + H]^+^	653.4 (y5^+^)	20	14
LHQLAFDTYQEFEEAYIPK^a^	781.4 [M + 2H]^2+^	993.4 (b16^2+^)	30	15
LHQLAFDTYQEFEEAYIPK^b^	781.4 [M + 2H]^2+^	1050.0 (b17^2+^)	30	15
LHQLAFDTYQEFEEAYIPK-SIL^a^	784.1 [M + 2H]^2+^	993.4 (b16^2+^)	30	15
LHQLAFDTYQEFEEAYIPK-SIL^b^	784.1 [M + 2H]^2+^	1050.0 (b17^2+^)	30	15

^a^Qualifier ion

^b^Quantifier ion

### Validation

The LC–MS/MS method was validated based on the guidelines for bioanalytical method validation of the Dutch Coordinating Commission for Quality Management in Medical Laboratories (CCKL) and the ISO 15189:2012 standard. The analytical response was the ratio of the peak areas found for the signature peptide of GH and its SIL internal standard, recorded using the LC–MS/MS signal of the quantifier ions (Table 1). The qualifier ions were included for the investigation of possible interferences. Analyse-it (v5.50) was used for Passing-Bablok, Bland–Altman analysis and linear regression for the matrix comparison test. Linearity of the method was determined by analyzing calibration curves on ten different days. Weighted linear regression was used with 1/*xx* as weighting factor. Furthermore, CV acceptance criteria were set at 15% for the concentrations and the slope (20% at the LLOQ concentration). The inter-assay variation was determined by analyzing the (human serum) low, medium, and high quality control (QC) samples in twofold on ten different days and the intra-assay variation by analyzing these QC samples in tenfold on the same day. The validity of analyzing samples after dilution was assessed by the analysis of a QC sample at 80 ng/mL after twofold dilution with rat plasma. The lower limit of the measuring interval (LLMI) was defined as the minimum analyte concentration quantified with adequate precision and established by analyzing four different human serum samples at concentrations near the lowest calibration standard in threefold on three different days. The accuracy of the method was tested by analyzing the low-, medium-, and high-QC samples, prepared in blank rat EDTA plasma with the WHO International Standard Somatropin and analysis in twofold on five different days. Spike recovery was determined for the medium- and high-QC samples from the inter-assay variation experiment and was calculated as follows: [(spiked concentration − unspiked concentration) / spiked concentration] × 100%. The interference of GHBP was tested by analysis of the low-QC human serum sample after the addition of a 100-fold molar excess of GHBP and incubation at 37 °C for 60 min (*n* = 3 on 1 day). Likewise, the interference of biotin was tested after the addition of 500, 750, 1000, or 1500 ng/mL biotin to the low-QC human serum sample. The interference from GH isoforms other than the target analyte GH1 (22 kDa) was assessed by analysis of a low QC human serum sample spiked with an additional 2.00 ng/mL of each isoform (GH1 20 kDa, GH2 22 kDa, and GH2 20 kDa). All samples for interference testing were analyzed in threefold on a single day. In addition to this isoform interference assessment, an isoform test was performed by spiking the low, medium, and high human serum QC samples with GH2 (22 kDa) at the same concentration as GH1 (22 kDa). A comparison of method performance for human serum, human EDTA plasma, and human heparin plasma was made by analyzing different mixtures of unspiked serum and plasma, each collected from two separate healthy individuals (and therefore each with two different GH1 (22 kDa) concentrations): 100% serum, 70% serum plus 30% plasma (v/v), 50% serum plus 50% plasma (v/v), 30% serum plus 70% plasma (v/v), and 100% plasma. The analytical behavior was concluded to be similar for plasma and serum if linear regression was the best fit for the concentration as a function of percentage of serum or plasma. The stability at − 80 °C and free/thaw stability of the stock solutions from both WHO and Prospec were determined against the corresponding freshly prepared stock solutions. In addition, the concentration of the stock solution from Prospec was established by comparing it with the WHO stock solution. The stability of endogenous GH1 (22 kDa) in human serum was assessed by storing freshly collected samples from two healthy individuals at + 4 °C and room temperature for 24 h or at − 20 °C and − 80 °C up to 27 days and comparing the results to those of the same fresh serum samples analyzed before storage (analysis in twofold) (see supplementary materials for more details). The freeze–thaw stability of these serum samples was determined in a similar way after three complete freeze–thaw cycles between − 20 °C or − 80 °C and room temperature. The stability of the signature peptides in the final serum extract was assessed by re-injection after storage for 72 h in the autosampler at 10 °C. Likewise, the stability in rat plasma (for use as calibrators) at spiked concentrations of 2.00 and 35.0 ng/mL recombinant GH1 (22 kDa) was assessed at − 20 °C and − 80 °C up to 133 days and after three freeze–thaw cycles. All bias and CV acceptance criteria were set at 15% (20% for LLMI), except for the stock stability assessment, for which 10% limits were used. The carry-over was determined by the analysis of alternating injections of low (2.0 ng/mL) and high (35.0 ng/mL) QC samples. The difference between the calculated mean concentration of the low QC samples and the calculated mean concentration of the low QC samples injected after high QC samples must be ≤ 3 times the standard deviation of the low QC samples. In addition to the QC sample, QC samples with concentrations of 15.0, 20.0, 25.0, and 30.0 ng/mL were included in the carry-over test in a similar way. As part of the validation, the LC–MS/MS method was compared to the IDS-iSYS human growth hormone immunoassay using 44 anonymized clinical samples.

## Results and discussion

### LC–MS/MS

**Fig. 3:**
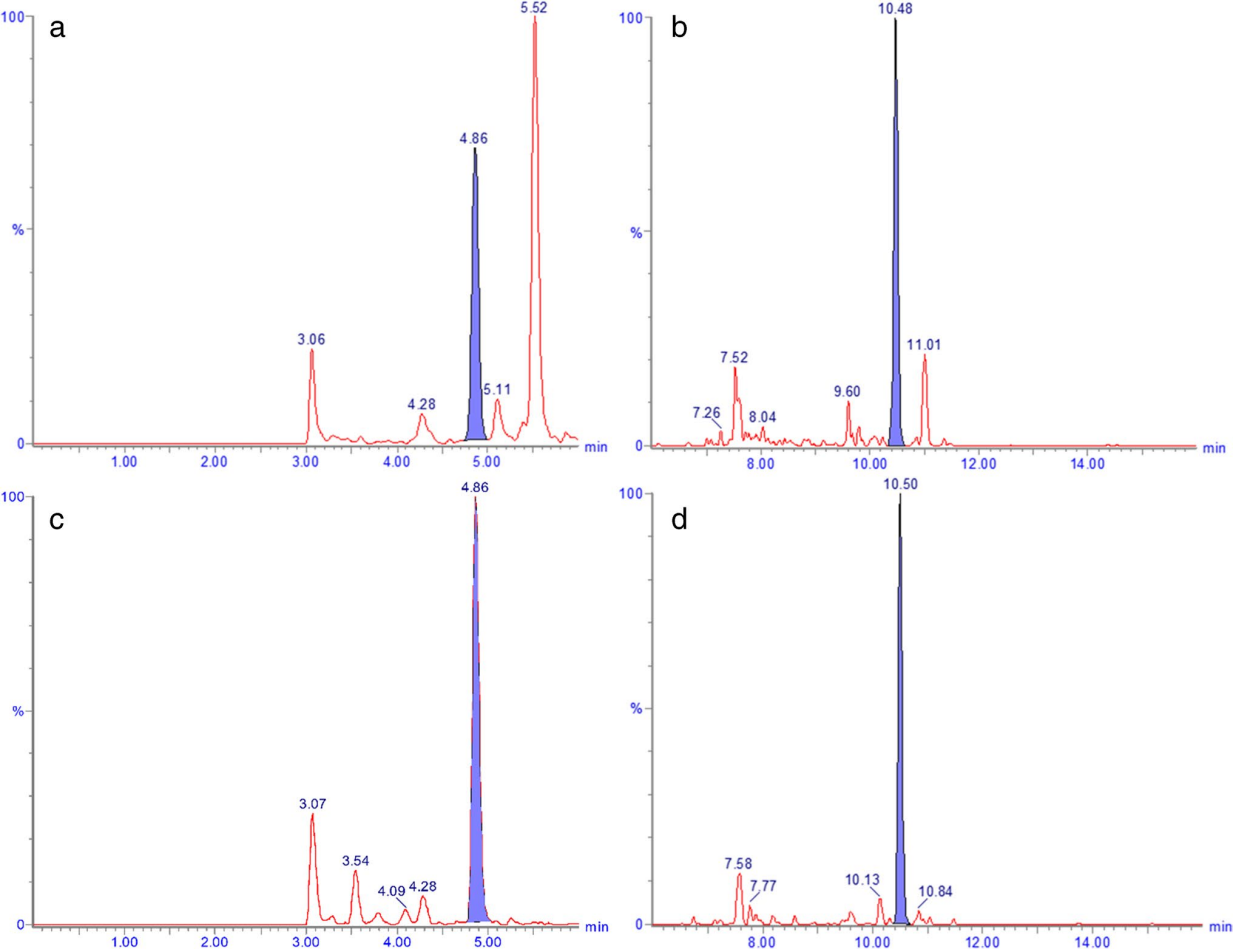
LC–MS/MS chromatograms of the GH1 (22 kDa) peptides SNLELLR (**a** and **c**) and LHQLAFDTYQEFEEAYIPK (**b** and **d**) recorded for a 0.5-ng/mL calibration standard in rat EDTA plasma (**a** and **b**) and a human serum sample with an endogenous concentration of 0.5 ng/mL (**c** and **d**)

The primary goal of this investigation was to develop a method for the selective quantification of GH1 (22 kDa), which is able to distinguish this major form from other GH isoforms and thus quantify it without any interference from the other forms. Secondly, it was deemed important to include a simultaneous readout for total GH, to allow a quick comparison in case of unexpected results. To differentiate GH1 (22 kDa) from other isoforms, a unique signature peptide for this GH isoform is needed, while a signature peptide that appears in all isoforms must be selected for the quantification of total GH. Since trypsin is the digestive enzyme of choice, because of its wide availability for a reasonable price, an in silico digestion of GH1 and the other isoforms was performed with this enzyme using mMass (http://www.mmass.org/) to generate the theoretically expected tryptic peptides. Out of the 18 theoretical peptides for GH1 (22 kDa), SNLELLR was selected for total GH because this peptide appears in all isoforms of GH, does not occur in any other endogenous human serum protein according the Basic Local Alignment Search Tool (BLAST) (https://blast.ncbi.nlm.nih.gov/Blast.cgi), and showed favorable stability and LC–MS properties. A second peptide, LHQLAFDTYQEFEEAYIPK, was selected for the quantification of GH1 (22 kDa) because this peptide is unique for this specific GH isoform according to BLAST. In addition, both peptides were confirmed not to occur in the entire rat plasma proteome, including rat growth hormone, which is a desirable feature because rat plasma was intended to be used as a proxy matrix to prepare calibration samples. For sufficient separation of the two signature peptides from endogenous matrix components, a slow gradient from 13 to 16% acetonitrile with an increase of 0.5% per minute was used for the elution of the SNLELLR peptide. For the elution of the second, more hydrophobic peptide, LHQLAFDTYQEFEEAYIPK, a quick step from 16 to 20% acetonitrile in 0.1 min was done, followed by a gradient of 1% acetonitrile per minute for another 5 min. With a subsequent 3-min column cleaning step at 95% acetonitrile and a 2-min re-equilibration at 13% acetonitrile, the total run time was 16 min per injection, which we considered reasonable for monitoring two tryptic peptides, and which is at least threefold faster than previously reported [11, 12, 15]. For the relatively small peptide, SNLELLR, a single protonated ion was selected in the first quadrupole, while for the larger LHQLAFDTYQEFEEAYIPK, this was a doubly protonated ion. With the mass transitions shown in Table 1, an optimized immunocapture step (see next section), and an 8-µL injection volume, sufficient sensitivity and selectivity were obtained to quantify both signature peptides at serum concentrations corresponding to 0.5–50 ng/ml of intact GH1 (22 kDa) (Fig. 3). This range covers the typical normal human endogenous concentration range.

### Immunocapture

For the quantification of proteins at low to sub-nanogram-per-milliliter concentrations in complex biological matrices, such as serum and plasma, a selective sample clean-up is necessary to reduce the interference from abundant plasma proteins. Generic techniques such as solid-phase extraction (SPE) based on ion-exchange or reversed-phase principles typically are not selective enough to eliminate matrix proteins before the digestion or the resulting peptides after digestion. These generic extractions lead to the occurrence of too many interfering peaks in the LC–MS chromatograms to allow reliable quantification of a protein analyte at the required concentrations [9]. Immunocapture (IC) approaches, based on the highly selective extraction of the protein of interest by an antibody, usually are a suitable alternative. For GH1 (22 kDa), an IC step was optimized with a commercially available mouse anti-human growth hormone monoclonal antibody (isoform specificity unknown). Using human serum spiked with the analyte at 100 ng/mL and quantification of the signature peptide SNLELLR after tryptic digestion of the IC extract, normalized by a SIL-peptide internal standard, the influence was tested of a number of important experimental parameters. Optimal results with regard to the amount of beads and antibody, temperature and duration of the capture, and elution steps are presented in Table 2. The final procedure resulted in a reproducible immunocapture recovery of 70%, which is comparable to the findings of Bults et al. with the same capture antibody for recombinant human GH [10]. The antibody also captured the GH2 isoform, albeit to a lower extent (30–60%). The sequence of combining GH, capture antibody, and magnetic beads was optimized to address the possible interference of biotin during IC [17–21]. Since streptavidin-coated beads are used to bind biotinylated capture antibody, endogenous biotin might compete with the antibody-GH complex for binding to streptavidin on the beads, if the serum sample would be mixed with the antibody solution before adding the beads. Therefore, the coupling between the biotinylated antibody and the streptavidin-coated beads should happen first to ensure that a maximum amount of capture antibody is already bound to the beads before the sample containing endogenous biotin is added. Another practical issue is the need to reduce the potential non-specific binding of GH1 22 (kDa) or other endogenous compounds to the sample preparation materials. Therefore, 0.1% of the detergent Tween-20 was included in the washing solutions. With this approach, the degree of non-specific binding of GH1 (22 kDa), assessed by processing high QC samples in triplicate without capture antibody, was 0.5%. To avoid interference of Tween-20 in the subsequent LC–MS assay, two final wash steps with 1 × PBS in water were added after IC and 10% acetonitrile was included in the elution solvent to enhance solubility of the signature peptides in the extract. Important for the robustness of the immunocapture step, and hence for the entire analytical method, is the batch-to-batch variability of the capturing efficiency of the commercially obtained antibody. This was tested by analyzing serum samples at the three QC concentrations using three different batches of the antibody, each in triplicate. Since all results agreed within 15% (supplementary Tables S20–S22), the capture antibody used in this method is sufficiently constant in quality to allow reproducible quantification of GH also when changing from one batch of the antibody to another.

**Table 2:** Overview of experimental parameters optimized for the immunocapture step

Parameter	Test range/condition	Optimum
Plate coating		
Concentration of antibody (µg/mL)	1/2/4/6	4
Amount of magnetic beads (µL)	10/15	10
Binding sequence	1. Mix antibody + GH (in serum sample), then add beads to capture the complex 2. Mix beads + antibody, then add GH (in serum sample)	2
Duration of incubation of antibody + beads (min)	60/120	120
Incubation temperature (°C)	37/45	37
Duration of incubation of antibody-bead complex + sample (min)	60/120	120
Capture temperature (°C)	37/45	37
Bead elution with 0.1 M citric acid in water:acetonitrile (90:10, v/v)		
Elution time (min)	10/20	10

### Digestion

**Fig. 4:**
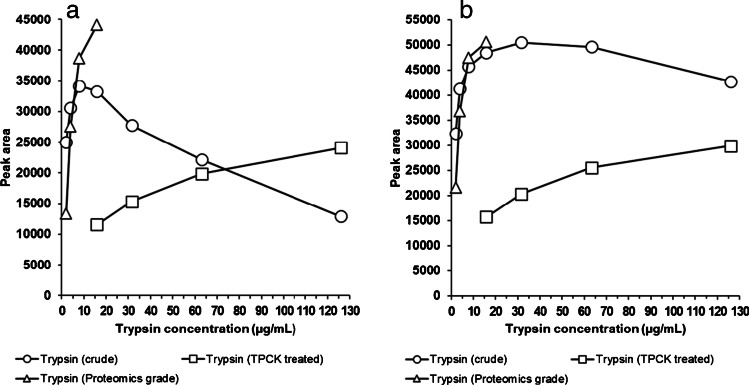
Optimization of the concentration of three types of trypsin for peptides LHQLAFDTYQEFEEAYIPK (**a**) and SNLELLR (**b**), 4 h of digestion after elution (*n* = 3)

**Fig. 5:**
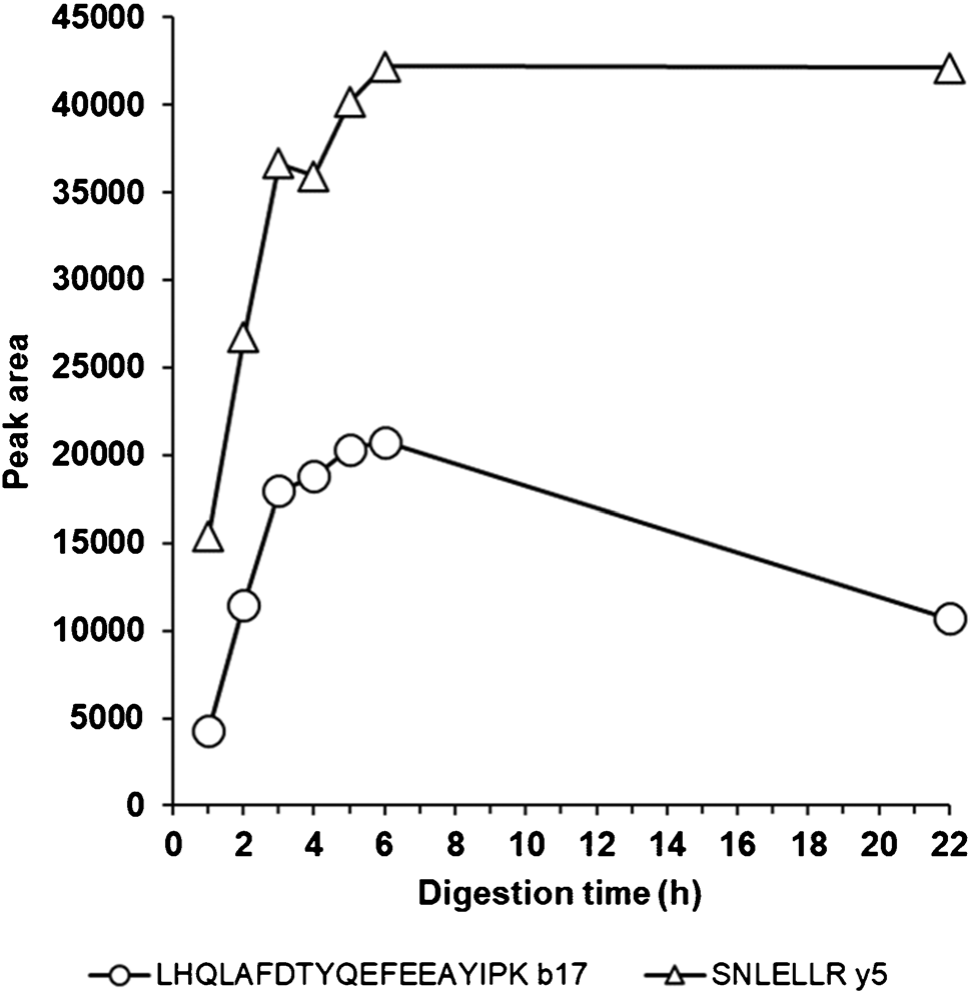
Optimization of digestion time for both signature peptides, 15.8 µg/mL of crude trypsin after elution (*n* = 3)

Important parameters to optimize for the digestion step are grade and concentration of the enzyme, digestion time, and the comparison between on-bead digestion directly after IC and digestion after elution of the protein analyte off the beads. Even though more proteases are available, trypsin is the most commonly used digestion enzyme because of its efficiency, specificity, and wide availability at a relatively low cost [22]. It has already proven its value for the digestion of GH1 (22 kDa) [9, 13, 14], and it was also selected for this investigation. The grade of trypsin is of importance for the speed of digestion and the occurrence of side reactions, but also determines the cost per analysis. Crude trypsin is by far the cheapest but shows residual chymotrypsin activity, meaning that the protein chain may be cleaved after an aromatic amino acid, such as tyrosine, phenylalanine, or tryptophan. Since the peptide LHQLAFDTYQEFEEAYIPK includes two tyrosines and two phenylalanines, it may be sensitive to degradation by this grade of trypsin. Therefore, two other products of the enzyme were also tested: purified proteomics-grade trypsin, which is free of chymotrypsin activity, but about 5000-fold more expensive than crude trypsin, and trypsin treated with the chymotrypsin inhibitor TPCK. Using serum spiked with 100 ng/mL of GH1 (22 kDa) and the optimized IC procedure for extraction, it was found that crude and proteomics-grade trypsin have approximately equal digestion activity per milligram of the enzyme, but that TPCK-treated trypsin is considerably less active (4-h digestion after elution, Fig. 4). A clear difference between crude and proteomics-grade trypsin is that the response for LHQLAFDTYQEFEEAYIPK decreased at crude trypsin concentrations above about 8 µg/mL, probably because of the further cleavage of the formed peptide by the higher level of chymotrypsin activity, while this effect was not seen for the proteomics-grade form. Peptide SNLELLR does not contain any aromatic amino acids, and as expected, no decreased response was encountered for higher concentrations of either of the types of trypsin. Increasing the concentration of crude trypsin above 15.8 µg/mL did not further improve the digestion efficiency for SNLELLR (4 h of digestion), indicating a maximum release of the peptide from GH is already obtained at this concentration of the enzyme. The same is likely true for the proteomics-grade trypsin, although this was not experimentally tested, because of the associated high costs. When a similar digestion (15.8 µg/mL of crude trypsin for 4 h) was performed directly on the beads, an up to fivefold lower recovery of the two signature peptides was obtained. This may be due to a more limited accessibility of GH for trypsin, when it is still bound to the capture antibody. Altogether, digestion with 15.8 µg/mL of crude trypsin after elution was considered a good compromise between digestion efficiency, stability, and costs. A final optimization of the digestion time showed maximum efficiency after 6 h of incubation, with a decrease in LHQLAFDTYQEFEEAYIPK response due of chymotrypsin activity only seen after overnight digestion (Fig. 5).

### Method performance

All individual validation results are included in the supplementary materials (Tables S1–S27). A summary is shown in Table 3 for both signature peptides.

**Table 3:** The maximum observed total bias and CV values for each validation experiment

Peptide	SNLELLR y5		LHQLAFDTYQEFEEAYIPK b17	
Validation item	Maximum bias (%)	Highest CV (%)	Maximum bias (%)	Highest CV (%)
Method characteristics				
Precision inter-assay	NA	4.5	NA	5.0
Precision intra-assay	NA	2.8	NA	5.9
Linearity	+ 1.2	4.2	+ 3.1	7.2
LLMI	NA	5.3	NA	9.1
Accuracy	+ 7.9	4.6	7.6	6.9
Interference of GHBP	− 4.3	2.1	+ 5.2	7.7
Interference of biotin (500 ng/mL)	+ 1.0	3.9	− 10.0	3.2
Interference of biotin (750 ng/mL)	− 2.2	0.8	− 7.4	4.2
Interference of biotin (1000 ng/mL)	− 11.4	3.9	− 14.7	1.5
Interference of biotin (1500 ng/mL)	− 22.2	2.5	− 22.8	2.8
Interference of isoforms	+ 73.2	5.5	− 0.2	4.1
Integrity of dilution	+ 2.1	1.3	+ 1.7	0.3
Stability of hGH-1 22 kDa in rat plasma				
Storage stability − 20/ − 80 °C (55 days/133 days)	− 8.8	7.6	− 5.6	12.3
Freeze–thaw − 20/ − 80 °C (3 cycles)	− 9.4	1.9	− 11.4	3.2
Stability of hGH-1 22 kDa in human serum				
Storage stability − 20/ − 80 °C (27 days)	− 5.4	8.4	− 4.3	6.1
Freeze–thaw − 20/ − 80 °C (3 cycles)	+ 4.8	1.7	− 4.8	2.7
Bench-top stability at room temperature (24 h)	+ 3.4	1.7	− 1.9	4.4
Storage stability + 4 °C (24 h)	+ 2.3	5.1	− 8.1	9.6
Stability of the signature peptides in final extract				
Autosampler 10 °C (72 h)	+ 2.6	2.8	− 2.0	6.7
Peptide	SNLELLR y5		LFDNAMLR y6	
Validation item	Bias (%)	CV (%)	Bias (%)	CV (%)
Stability of hGH-1 22 kDa in stock solution				
Frozen storage – 80 °C (WHO) (733 days)	+ 3.7	1.8	+ 6.3	0.9
Frozen storage – 80 °C (Prospec) (193 days)	− 4.2	1.0	− 3.6	0.8
Freeze–thaw – 80 °C (WHO) (5 cycles)	− 0.6	1.4	+ 2.7	1.2
Freeze–thaw – 80 °C (Prospec) (6 cycles)	− 6.1	1.6	− 4.8	1.4
Peptide	SNLELLR y5		LHQLAFDTYQEFEEAYIPK b17	
Validation item	Mean (%)	CV (%)	Mean (%)	CV (%)
Method characteristics				
Spike recovery QC Med	102	4.8	102	5.1
Spike recovery QC High	95	4.8	94	5.3

### Precision and accuracy

For both signature peptides, the calibration curves (*n* = 10) were linear across the calibration range of 0.5–50 ng/mL with correlation coefficients (*R*^2^) > 0.99, and accuracy and precision of the calibrators below 4% bias and 8% CV, respectively. Method precision for low endogenous GH serum level (established as 2.16 ng/mL and 2.05 ng/mL for SNLELLR and LHQLAFDTYQEFEEAYIPK, respectively) and for this serum spiked with an additional 10.0 or 35.0 ng/mL recombinant GH1 (22 kDa), all analyzed against a calibration curve in rat plasma, was acceptable (CV < 6%) for both signature peptides and for both inter-day (*n* = 2 on 10 days) and intra-day (*n* = 10 on 1 day) analyses. Integrity of (twofold) dilution of patient samples with rat plasma was demonstrated for both peptides (bias < 3% and CV < 2%). The lower limit of the measuring interval was very close to the lowest calibration level at 0.682 ng/mL and 0.542 ng/mL for SNLELLR and LHQLAFDTYQEFEEAYIPK, respectively, with a CV below 10% found for both signature peptides. Method accuracy, assessed using rat plasma spiked with the WHO standard Somatropin at 2.00, 10.0, and 35.0 ng/mL, against calibration curves containing the regular GH1 (22 kDa) reference standard, was acceptable with values for bias below 8% (*n* = 2 on 5 days) for both signature peptides. This demonstrates that the reference standard used allows direct comparison of results with other methods that are normalized against the WHO Somatropin standard. Mean spike recovery was determined at two concentrations to assess accuracy in human serum and ranged from 94 to 102% for the two signature peptides.

### Selectivity and interference

Method reliability is not impacted by binding of GH to the binding protein GHBP, as demonstrated by acceptable results (< 8%) for CV (precision) and bias (accuracy) in the presence of a 100-fold molar excess of GHBP, compared to analysis in the absence of GHBP. This shows that GHBP binds GH at a position that does not hinder capture of the GHBP-GH complex by the antibody, or sufficient dissociation of the complex occurs to allow GH to be captured. Similarly, addition of up to 1000 ng/mL biotin was found not to affect the measurement in an unacceptable way (bias < 15% and CV < 4%). The presence of 1500 ng/mL biotin, however, did lead to a negative bias of around 23%, possibly by displacement of the biotinylated capture antibody-GH complex from the streptavidin-coated beads by biotin. Since normal concentrations of biotin typically range from 0.1 to 0.8 ng/mL [18, 21], with an increase to 1160 ng/mL having been reported [17] when patients take biotin-containing supplements, this effect is only expected to be relevant in rare and extreme cases [20]. The result for GH1 (22 kDa) as represented by peptide LHQLAFDTYQEFEEAYIPK at an endogenous serum concentration of 2.05 ng/mL was not affected by the presence of an additional 2.00 ng/mL of the three other GH isoforms, which confirms the suitability of this peptide for selectively quantifying the major form of GH. The corresponding result for the common peptide SNLELLR showed an extremely high positive bias of 73.2%, caused by the (partial) co-extraction of the other isoforms from the serum sample and release of this signature peptide upon their digestion. This shows that the capture antibody used also binds one or more of these GH isoforms and is thus not specific for the GH1 (22 kDa) form. Likewise, peptide LHQLAFDTYQEFEEAYIPK was not affected either by the addition of 2.00, 10.0, and 35.0 ng/mL of GH2 (22 kDa) to the low, medium, and high QC samples, respectively. In this case the SNLELLR peptide showed positive biases of 67.5%, 32.5%, and 31.9% for QC low, medium, and high respectively. Apparently, co-extraction of GH2 (22 kDa) decreases at higher concentrations, which may be due to a more pronounced competition between GH1 (22 kDa) and GH2 (22 kDa) for binding to the capture antibody.

### Sample type

The equivalence of serum and plasma as the sample matrix for the quantification of GH1 (22 kDa) was assessed by analyzing different mixtures of a serum and a plasma sample, with percentages ranging from 100% serum to 100% plasma. Figs. S2 (EDTA plasma) and S3 (heparin plasma) present the GH1 (20 kDa) concentrations found in the different mixtures for peptide LHQLAFDTYQEFEEAYIPK, and Figs. S4 (EDTA) and S5 (heparin) for peptide SNLELLR, and they show a good correlation (linear fit with *R*^2^ > 0.99). Human EDTA and heparin plasma can, therefore, also be used as the sample matrix instead of human serum.

### Stability and carry-over

GH1 (22 kDa) is sufficiently stable in stock solution during storage at − 80 °C (193 days, and even up to 733 days for the WHO standard) and over six freeze/thaw cycles between − 80 °C and room temperature (five for the WHO standard). Furthermore, adequate stability was demonstrated in human serum at − 80 °C and – 20 °C (at least 27 days) and after three freeze/thaw cycles between − 80 or − 20 °C and room temperature. Similar results were found for storage of GH1 (22 kDa) in rat plasma, which confirms the suitability for long-term use of spiked rat plasma for calibration. In addition, GH1 (22 kDa) showed no stability issues in human serum when stored at + 4 °C and room temperature for 24 h. Finally, processed samples remain stable up to 72 h when placed in the autosampler at 10 °C. Carry-over was acceptable for peptide SNLELLR in all situations and for peptide LHQLAFDTYQEFEEAYIPK at concentrations up to 30 ng/mL. Only for the high QC concentration (35 ng/mL) was carry-over to a subsequent sample unacceptable, at 5.5 times the standard deviation for the low QC sample, where ≤ 3 is allowed. Since this peptide is significantly more hydrophobic than the peptide SNLELLR, it is more prone to adsorption, even though the LC–MS system was flushed 3 min at 95% acetonitrile. Care should thus be taken in evaluating samples with a concentration below 2 ng/mL, which were injected into the analytical system directly after a sample with a concentration above 30 ng/mL, a situation which does not frequently occur in clinical practice. Since carry-over is strongly instrument dependent, it is advisable that this effect be investigated for each system that is used for GH analysis.

### Comparison with a GH immunoassay

**Fig. 6:**
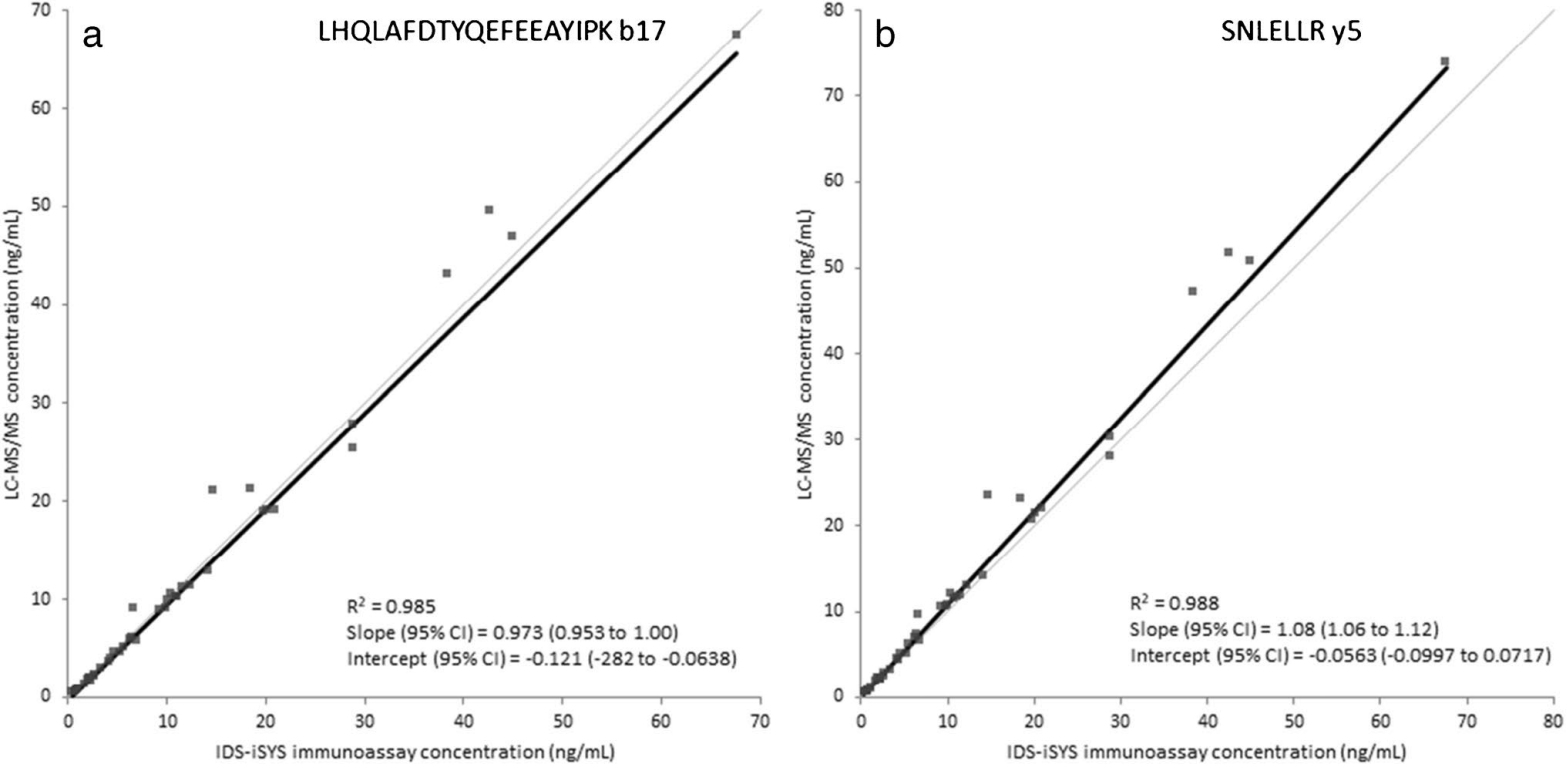
Passing-Bablok results for the comparison of the LC–MS/MS method for GH1 (22 kDa) (**a**) and for total GH (**b**) with the IDS-iSYS immunoassay

**Fig. 7:**
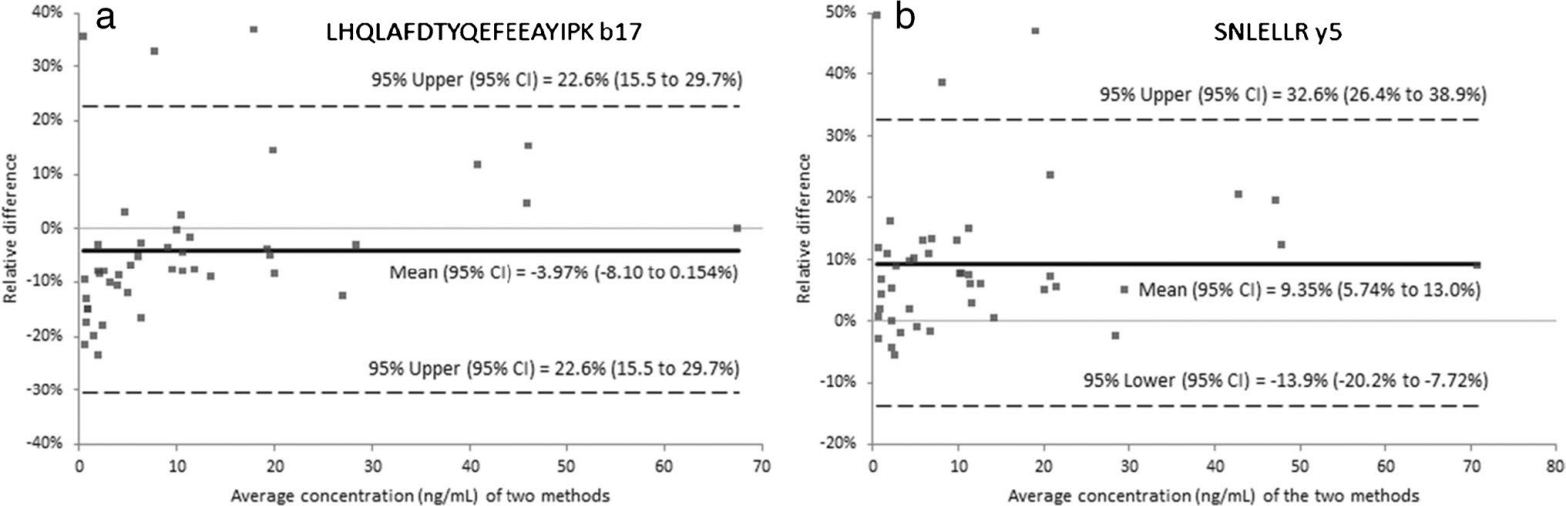
Bland–Altman plots for the comparison of the LC–MS/MS method for GH1 (22 kDa) (**a**) and for total GH (**b**) with the IDS-iSYS immunoassay

A comparison was made between the LC–MS/MS method and the IDS-iSYS GH immunoassay by analyzing 44 clinical serum samples with both methods. Passing-Bablok regression data and Bland–Altman plots are shown in Fig. 6 and Fig. 7, respectively. Based on the Passing-Bablok regression for both peptides, the methods correlate well (*R*^2^ > 0.98). For peptide LHQLAFDTYQEFEEAYIPK, the slope is 0.973 with the 95% confidence interval of the slope being between 0.953 and 1.00 (and therefore within the acceptable range of 0.9 to 1.1), and the *y*-intercept includes the origin. This shows a good correspondence between both methods and implies that not only the LC–MS/MS method but also the immunoassay has a good selectivity towards the GH1 (22 kDa) isoform. For the SNLELLR peptide, the slope is 1.08 with a 95% confidence interval of 1.06 to 1.12. This confirms that the LC–MS/MS method for this peptide gives significantly higher concentrations than the immunoassay, which must be due to the presence of other GH isoforms in the serum samples which are not detected by the immunoassay, but do generate a response in the LC–MS/MS signal for the general peptide. This is confirmed by the Bland–Altman plots for both signature peptides, in which the total GH response expressed by SNLELLR has an average positive bias of 9.4% relative to the mean concentration of both methods. Comparison of the LC–MS/MS results for both peptides (Supplementary Fig. S1) shows a good correlation with an average positive bias of 13% for SNLELLR compared to LHQLAFDTYQEFEEAYIPK. This further supports the occurrence of other GH isoforms in serum, which do contain the SNLELLR sequence but not the GH1 (22 kDa) specific LHQLAFDTYQEFEEAYIPK. The validation samples containing additional GHBP, biotin, or the other three GH isoforms were also analyzed using the IDS-iSYS immunoassay. The results were not affected by the presence of the isoforms or a 100-fold excess of GHBP, but the sample spiked with the lowest concentration (500 ng/mL) of biotin already showed a huge negative bias of > 95%. This means that the immunoassay performs acceptably in the presence of binding protein and other GH isoforms, but is seriously affected in case of high biotin concentrations.

### Analysis of serum from late pregnancy 

As a further test of the selectivity of the LC–MS/MS method, serum samples from five individuals, who were in the 29^th^ to 37^th^ week of their pregnancy, were analyzed and the results of both peptides were compared. In the later part of pregnancy, circulating GH1 is completely replaced by GH2, with peak concentrations after 35 to 37 weeks [23–25]. The peptide specific for GH1 (22 kDa) was unquantifiable (< 0.5 ng/mL) in all samples, while for the SNLELLR peptide, concentrations between roughly 60 and 120 ng/mL were found (after up to threefold dilution of the samples with rat plasma to bring the concentrations within the range of the calibration curve). Apparently, no GH1 (22 kDa) is present in these samples, which confirms literature findings using GH2 specific antibodies and further demonstrates the selectivity of the LC–MS approach. A relatively high concentration of one or more other GH-related proteins appears to be present in the serum of these pregnant women. Although it cannot be established with the current method which isoforms are actually there, it is not unlikely that GH2 and HCS, which are known to contain the sequence SNLELLR, are responsible for the response.

### Conclusion

The LC–MS/MS method presented here is able to selectively quantify the major GH isoform, GH1 (22 kDa), in human serum and differentiate it from the other circulating isoforms at the clinically relevant concentrations between 0.5 and 50 ng/mL. The satisfactory results of a thorough method validation in terms of precision, accuracy, selectivity, and stability, and the good batch-to-batch reproducibility of the critical antibody reagent demonstrate the capability of this method for the unambiguous determination of this GH isoform over longer periods of time. With its sample volume of 100 µL and its LC–MS/MS run time of 16 min, it is well suited for the analysis of larger batches of samples, and although the required immunocapture step and subsequent digestion are relatively time-consuming, in practice, a throughput of 60 samples per day can be routinely achieved. Although the IDS-iSYS immunoassay also performs acceptably for GH1 (22 kDa) when no high levels of biotin are present in the samples, and this platform is probably preferred for routine analysis, we believe that the LC–MS/MS method has added value for specialized laboratories in which GH disturbances are investigated, e.g., to give more clarity in case results obtained with routine immunoassay analyzers are ambiguous or conflicting, and for use in routine laboratory practice. In addition, the fact that the method also provides a readout for total GH is helpful in resolving possible conflicting measurement results in patients as well as between different GH immunoassays, ultimately supporting optimal patient care.

## Supplementary Information

Below is the link to the electronic supplementary material.

Supplementary file1 (DOCX 2455 KB)
