# Supplementary material for: Selective quantification of the 22-kDa isoform of human growth hormone 1 in serum and plasma by immunocapture and LC–MS/MS
Source: Anal Bioanal Chem. 2022 Jul 15;414(20):6187–200. doi: 10.1007/s00216-022-04188-z (PMC9314277; doi:10.1007/s00216-022-04188-z)
Supplement: Supplementary file 1 — Supplementary file1 (DOCX 2455 KB) [file 216_2022_4188_MOESM1_ESM.docx]

# Supplementary materials

The calculation of average, bias and coefficient of variance (CV) values was performed before rounding. All values were taken from the Waters Targetlynx 4.1 data-processing sofware.

Table S1. Individual calibration curve results.

Table S2. Inter-assay precision results.

Table S3. Spike recovery results.

Table S4. Intra-assay precision results.

Table S5. Accuracy results.


Table S6. Integrity of dilution results.

Table S7. LLMI results.

Table S8. Autosampler stability results at 10 °C.

Table S9. Storage stability results in rat plasma at -80 °C.


Table S10. Storage stability results in rat plasma at -20 °C.

Table S11. Freeze/thaw stability results in rat plasma -80 °C.

Table S12. Freeze/thaw stability results in rat plasma -20 °C.

Table S13. Storage stability results in human serum at t=0.

Table S14. Storage stability results in human serum at -80 °C.


Table S15. Storage stability results in human serum at -20 °C.

Table S16. Freeze/thaw stability results in human serum -80 °C.

Table S17. Freeze/thaw stability results in human serum -20 °C.

Table S18. Storage stability results in human serum at +4 °C.

Table S19. Bench-top stability results of human serum at room temperature.

Table S20. Antibody batch comparison. Batch 1 is set as reference.


Table S21. Antibody batch comparison. Batch 2 is compared with batch 1.

Table S22. Antibody batch comparison. Batch 3 is compared with batch 1.


Table S23. Biotin interference test results.

Table S24. GBHP interference test results.

Table S25. Isoform interference test results.

Table S26. Isoform test results.

Table S27. Stock stability test results.


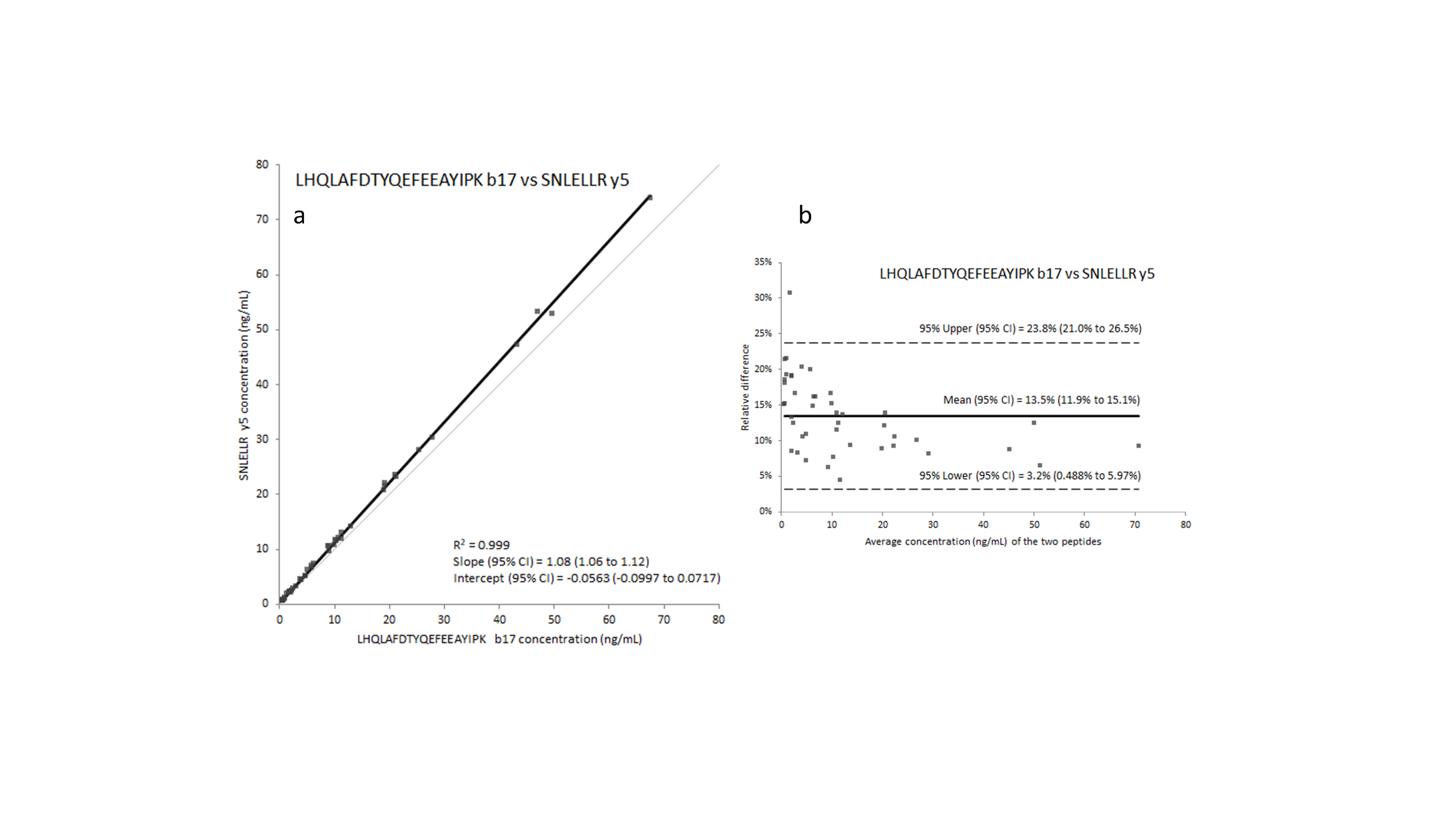


Figure S1. Comparison between the two different peptides using Passing-Bablok (a) and Bland-Altman plots (b).


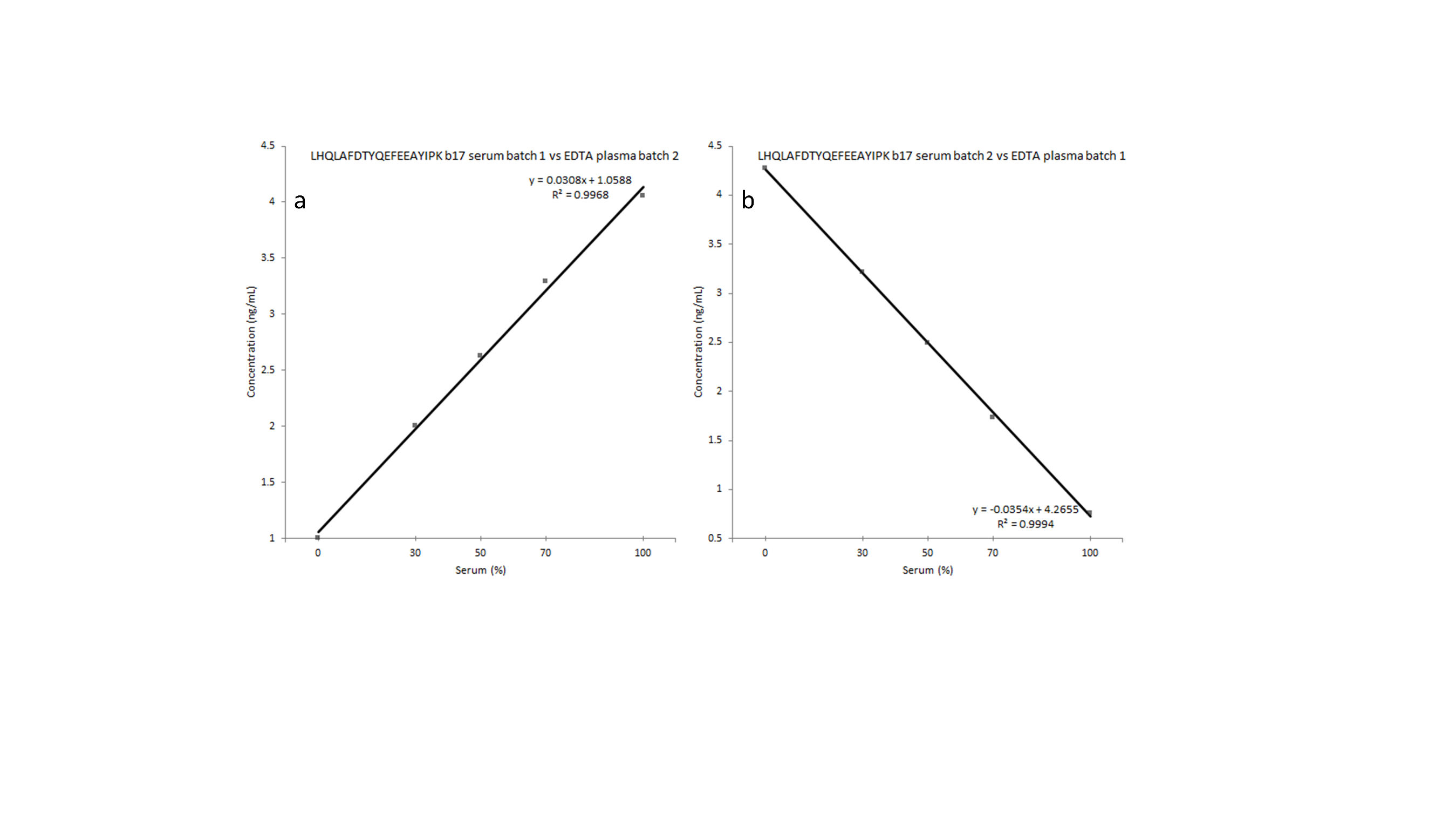


Figure S2. Matrix comparison for peptide LHQLAFDTYQEFEEAYIPK. Serum batch 1 vs EDTA plasma batch 2 (a) and serum batch 2 vs EDTA plasma batch 1 (b).


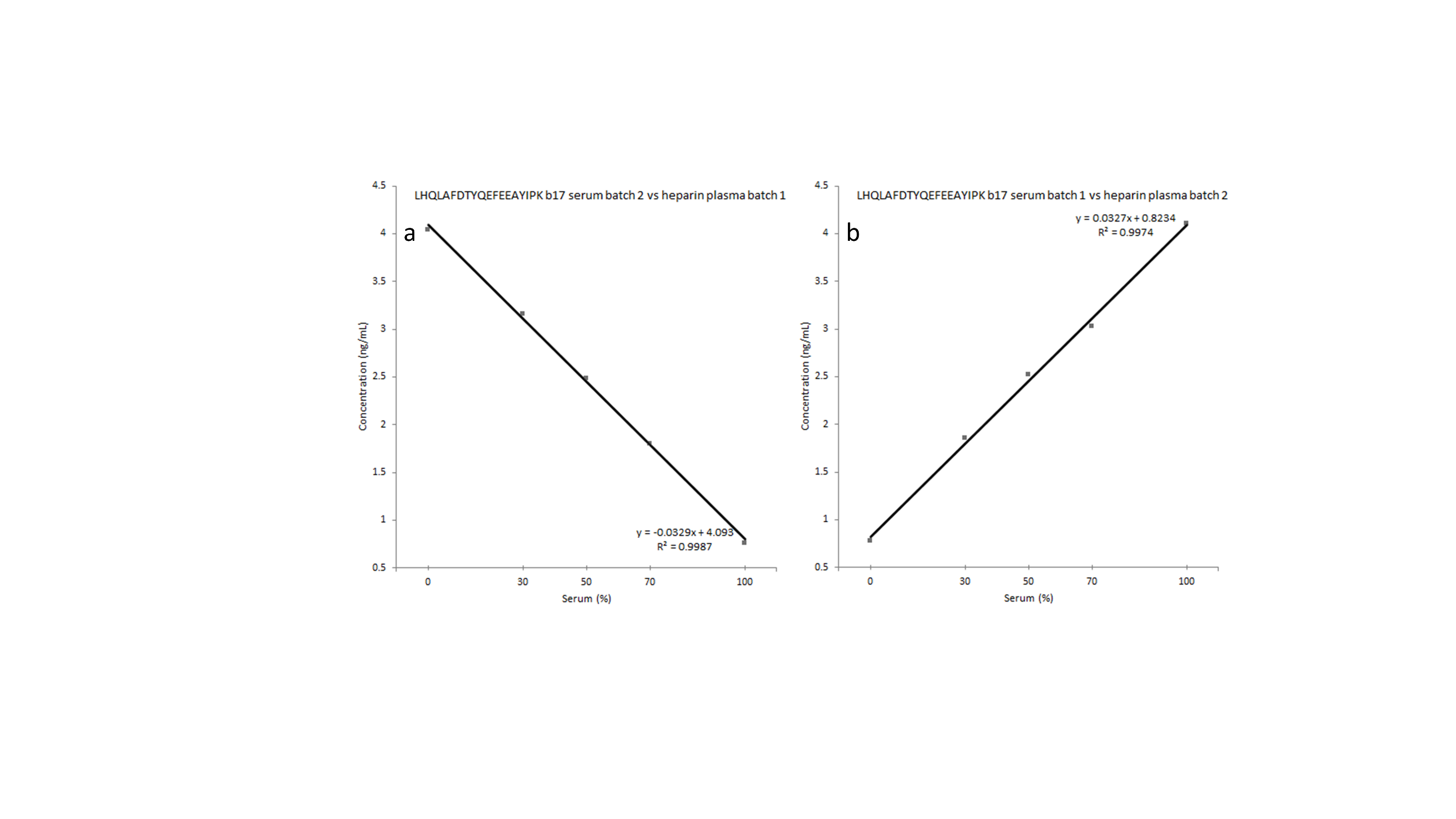


Figure S3. Matrix comparison for peptide LHQLAFDTYQEFEEAYIPK. Serum batch 1 vs heparin plasma batch 2 (a) and serum batch 2 vs heparin plasma batch 1 (b).


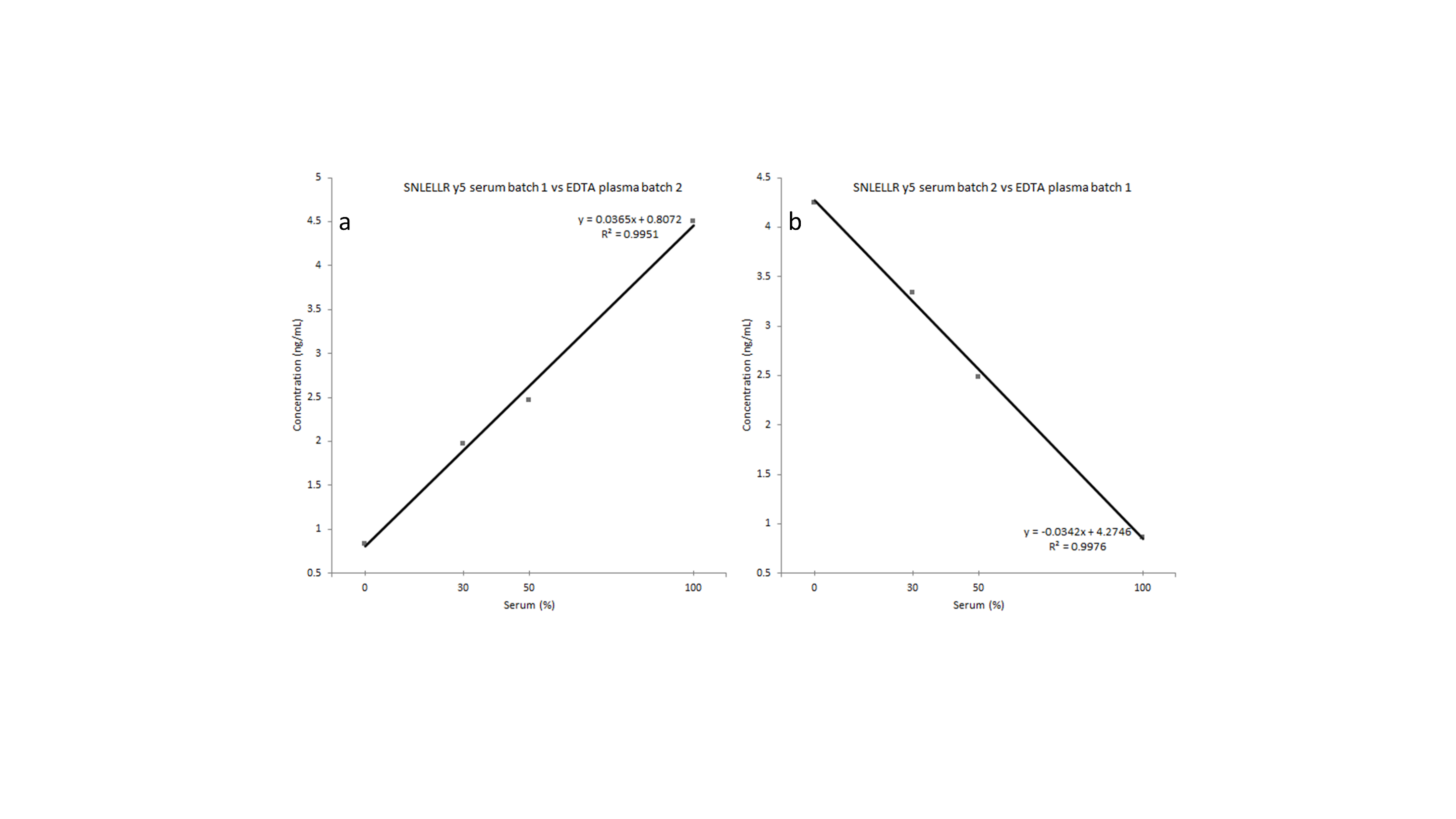


Figure S4. Matrix comparison for peptide SNLELLR. Serum batch 1 vs EDTA plasma batch 2 (a) and serum batch 2 vs EDTA plasma batch 1 (b).


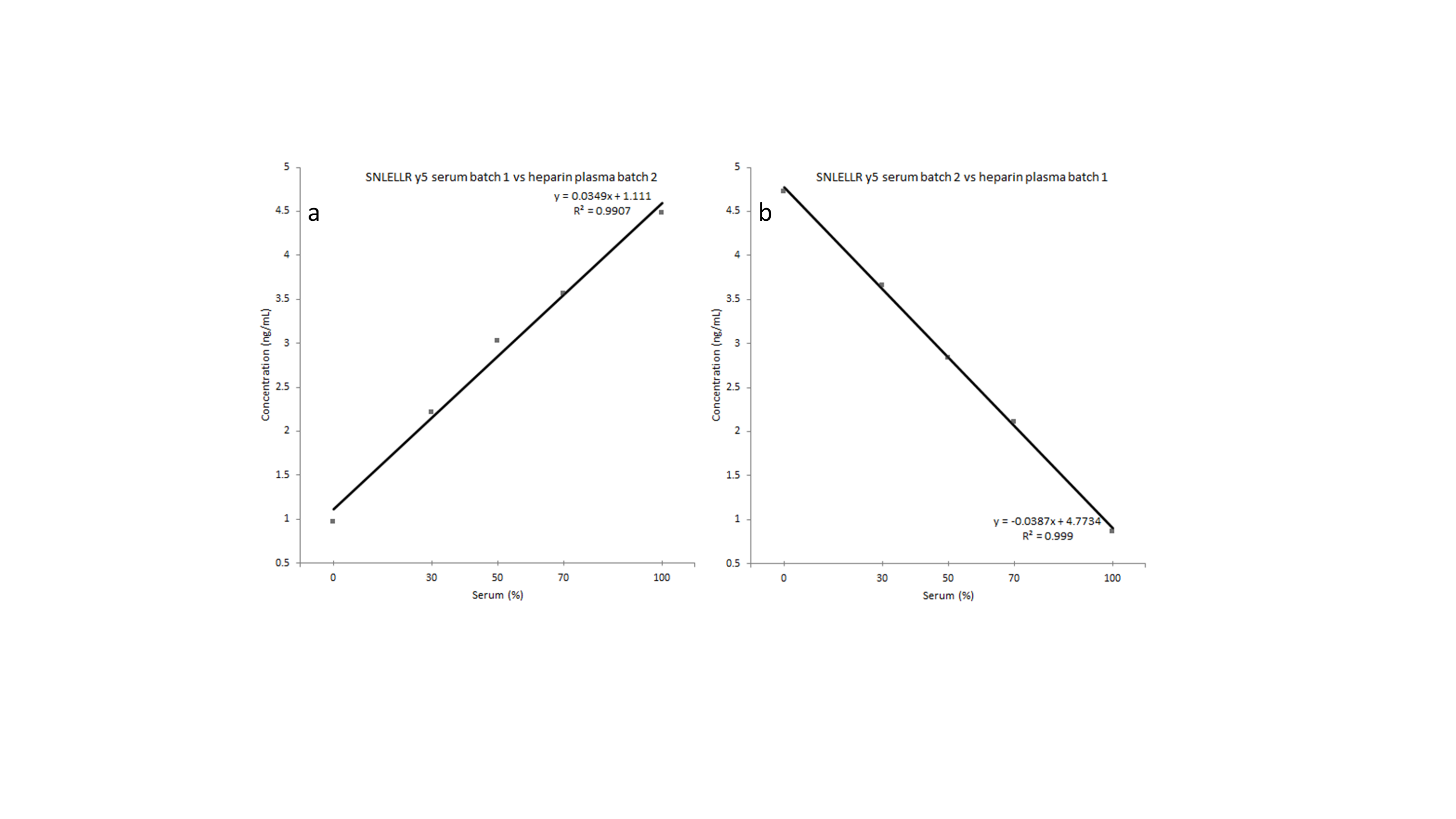


Figure S5. Matrix comparison for peptide SNLELLR. Serum batch 1 vs EDTA plasma batch 2 (a) and serum batch 2 vs EDTA plasma batch 1 (b).
